# Multiplexed optical fiber cell temperature sensing system with high sensitivity and accuracy

**DOI:** 10.1117/1.JBO.28.4.047001

**Published:** 2023-04-08

**Authors:** Yu Guo, Yanxia Shen, Xinyu Sun, Shulin Song, Haodong Wu, Xiulan Sun

**Affiliations:** a Jiangnan University, School of Internet of Things Engineering, Wuxi, China; b Jiangnan University, School of Food Science and Technology, Collaborative Innovation Center of Food Safety and Quality Control, State Key Laboratory of Food Science and Technology, Wuxi, China; c Nanjing University, School of Physics, Nanjing, China

**Keywords:** optical fiber sensing system, beat frequency signal, fiber Bragg grating, two-channel cell temperature sensing

## Abstract

**Significance:**

A multiplexed fiber laser sensing system for cell temperature is proposed. To the best of the authors’ knowledge, this is the first multilongitudinal mode (MLM) optical fiber laser sensor array designed for cell temperature sensing.

**Aim:**

A two-channel cell temperature sensing system with high sensitivity and real-time sensing capability is achieved. The temperature change of human hepatoellular carcinomas (HepG2) cells under the influence of exogenous chemical aflatoxin B1 (AFB1) can be monitored in real time.

**Approach:**

A fiber laser cavity consists of a pair of fiber Bragg gratings (FBGs) with matched central wavelengths and a piece of erbium-doped fiber (EDF). The static FBG is utilized for design of fiber laser cavity and laser modes selection. In comparison, the sensing FBG is used for cell temperature sensing. The sensing FBG has a length of 10 mm and a diameter of 200  μm. Beat frequency signals (BFS) are generated by MLM lasers after optical-to-electrical conversion at a photodetector. Frequency change of a BFS is closely related to the reflected wavelength change of the sensing FBG. Through frequency division multiplexing, two fiber laser cavities are designed in the sensing system for two-channel temperature sensing. Frequency shift of a BFS that represents temperature change of cells can be automatically recorded in seconds.

**Results:**

A two-channel cell temperature sensing system is designed with high sensitivities of 101.62 and 119.82  kHz/°C, respectively. The temperature change of HepG2 cells under the influence of exogenous chemical AFB1 is monitored in real time.

**Conclusions:**

The proposed system has the advantages of simple structure, high sensitivity, and two-channel sensing capability. Our study provides a simple and effective method to design a fiber laser sensor system without complex demodulation techniques and expensive optical components.

## 1 Introduction

Temperature plays an important role in the development of life. A series of physiological activities in cells are accompanied by the exchange and release of energy, which can be manifested as temperature changes.^1^ In order to achieve highly accurate temperature measurement of body, tissue, or cell, the capability of temperature determination in a compact region has to be achieved by the sensing system. In addition, a high sensing accuracy is also required by the system. Therefore, researchers have done a lot of research work, and different techniques have been proposed. Several different techniques have been reported including multichannel isothermal microcalorimeter,^2^ passive microwave radiometer technique,^3^ near-field microwave radiometer,^4^ infrared thermography,^5^ optical thermometers based on fluorescent organic molecules or rare earth–doped nanomaterials,^6^^–^^10^ and fiber Bragg grating temperature sensors.^11^^–^^13^

Isothermal microcalorimetric techniques can have higher reproducibility and detectability than many other analytical methods. However, the low rate of sample throughput has limited their use in practical work. To some extent, this disadvantage has to be balanced by the utilization of multichannel instruments. So that several samples can be measured simultaneously.^2^ In Ref. 2, a 48-channel isothermal microcalorimeter is presented that can measure 48-well microtiter plate with size larger than $72\times 54\;{\mathrm{mm}}^{2}$. This method cannot work very well for temperature sensing of small amount of samples.

Microwave radiometer technique is a noninvasive temperature measurement technique, which is widely used in cancer hyperthermia. Temperature of deep tissue can be detected by this kind of technique. However, the microwave technique is easy to be disturbed by electromagnetic signals and the application conditions are strict.^3^^,^^4^ In Ref. 3, a biconical patch antenna with size of $40\times 5\;{\mathrm{mm}}^{2}$ is utilized for temperature measurement. In Ref. 4, a circular patch probe with size of $4\times 4\;{\mathrm{cm}}^{2}$ is utilized as skin-mounted probe for temperature sensing. The infrared thermography technique, which is represented by infrared thermal imager, is a fast, noninvasive, and noncontact method. It can directly display the temperature distribution of the measured part. This method is widely utilized in practical life, but it is subject to the limitation of light penetration. Therefore, it is only suitable for surface temperature detection. An IR camera (FLIR SC 4000) and a small cartridge heater with a length of 12.7 mm and a diameter of 3.1 mm were utilized for the temperature sensing.^5^ For the optical thermometers based on fluorescent organic molecules or rare earth doped nanomaterials, noncontact optical temperature sensing can be realized. However, fluorescence lifetime, fluorescence intensity, and fluorescence intensity ratio are all highly sensitive to temperature. In addition, utilizing fluorescence lifetime or intensity to determine temperature is strongly dependent on excitation source, optoelectronic system, background noise, and other factors. Therefore, it is difficult to guarantee accuracy and repeatability of temperature measurement.^6^

In comparison, optical fiber sensing system especially fiber Bragg grating (FBG) sensing system has attracted lots of attention for biological temperature measurement due to its antielectromagnetic interference, compact size, and high sensitivity. A linearly chirped FBG with a length of 15 mm and a diameter of 200  μm has been used as a temperature sensor for online monitoring of radiofrequency thermal ablation (RFTA).^11^ A fiber Bragg grating-based temperature sensing probe was designed for distributed temperature measurement of tissue undergoing laser ablation.^12^ The FBG sensor’s length is only 3 mm. Fiber-optic sensors are integrated with commercial medical instruments for temperature monitoring during tumor RFTA treatments.^13^ The heat propagation over the area under treatment can be measured by five FBG sensors properly bounded to the RFTA probe. A spatial resolution of 5 mm is achieved by the method. For the aforementioned FBG-based temperature sensing systems, optical spectrum analyzer (OSA) has to be utilized to determine the temperature change. The temperature is measured by monitoring corresponding FBG’s wavelength shift. The requirement of an expensive OSA has the problems of big size, long scanning time, and low-temperature sensitivity.

Optical fiber laser sensing system based on beat frequency signal (BFS) provides a new sensing method. BFSs are generated by multilongitudinal mode (MLM) lasers after optical-to-electrical conversion at a photodetector (PD). Extremely high sensing sensitivity is observed by monitoring BFS’s frequency change instead of laser’s wavelength shift.^14^ Therefore, OSA is not required for multilongitudinal mode fiber laser sensor (MLMFLS) system. The sensing system has been utilized for detection of many physical parameters, such as vibration, temperature, strain, and refractive index.^15^^–^^17^ Dual-parameters sensing, such as strain, temperature, and load, can be realized by monitoring BFS.^18^^,^^19^ Multiplexed MLMFLS arrays are demonstrated with good performances for use in large-scale monitoring fields.^20^^–^^23^

In this paper, a multiplexed MLM fiber laser sensor system for cell temperature sensing is proposed. To the best of the authors’ knowledge, this is the first two-channel cell temperature sensing system ever reported by an MLMFLS system. A fiber laser cavity consists of a pair of common FBGs with matched central wavelengths and a piece of erbium-doped fiber (EDF). The static FBG is utilized for design of fiber laser cavity and laser modes selection. In comparison, the sensing FBG is used for cell temperature sensing. The sensing FBG has a length of 10 mm and a diameter of 200  μm. BFSs are generated by MLM lasers after optical-to-electrical conversion at a PD. Frequency change of a BFS is closely related to the reflected wavelength change of the sensing FBG. By frequency division multiplexing, two fiber laser cavities are designed in the sensing system for simultaneous temperature sensing of two different channels. By utilizing Python program to control platinum resistance thermometer and real-time spectrum analyzer (RSA), the BFS’s frequency shift can be automatically recorded in seconds. In order to achieve an accurate demodulation of the BFS’s frequency shift, a new method of finding the BFS’s center point is utilized, which can avoid the error caused by large frequency jitter. By utilizing the designed sensing system, the temperature change of human hepatoellular carcinomas (HepG2) cells under the influence of exogenous chemical aflatoxin B1 (AFB1) is monitored in real time. Experiments demonstrated the sensing system’s high accuracy, sensitivity, and two-channel temperature sensing capability.

## 2 Principle

**Fig. 1:**
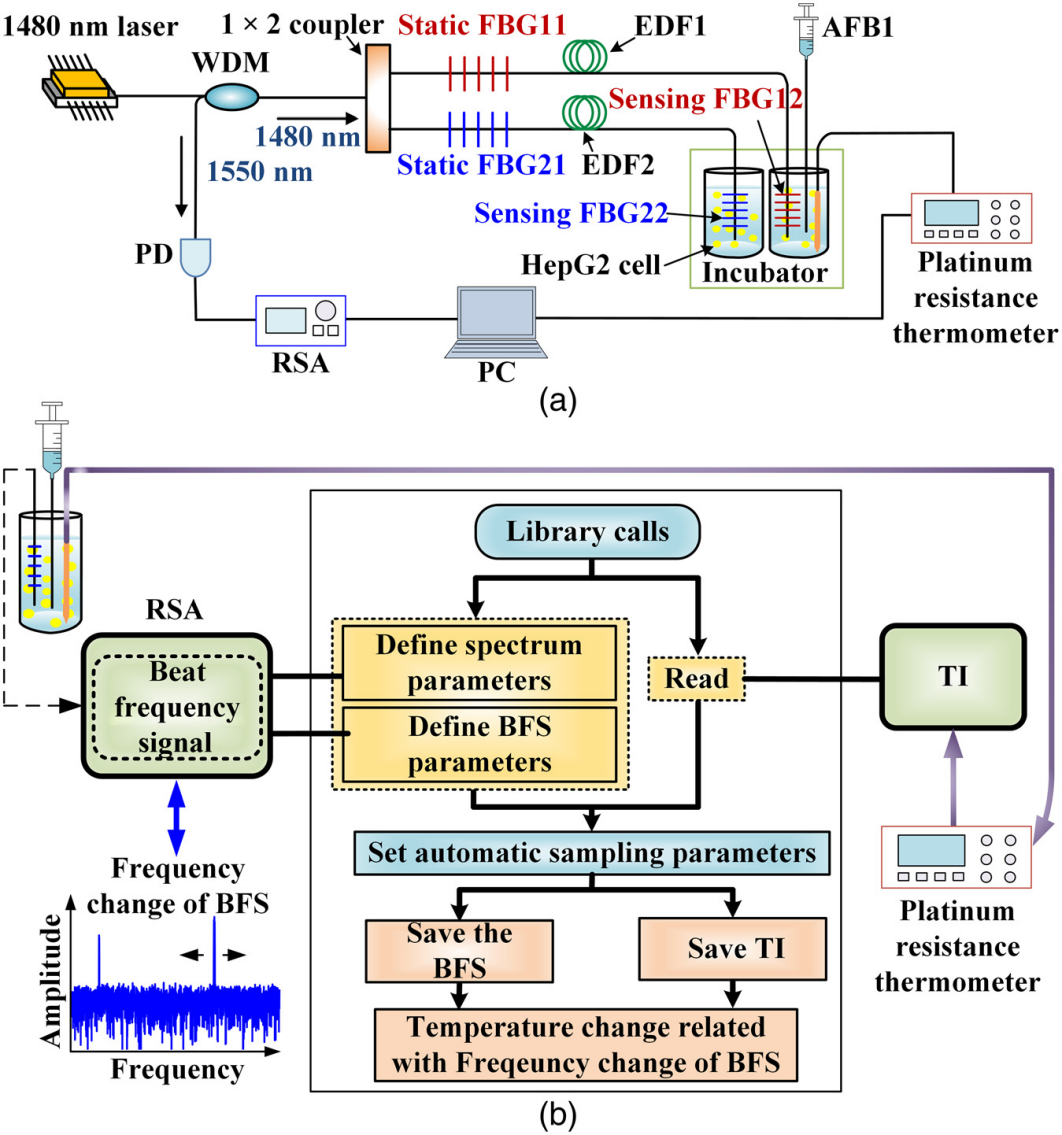
(a) Schematic configuration of the proposed multiplexed fiber cell temperature sensing system. (WDM, wavelength division multiplexer; FBG, fiber Bragg grating; EDF, erbium-doped fiber; PD, photodetector; RSA, real-time spectrum analyzer; PC, personal computer; AFB1, aflatoxin B1; and HepG2, human hepatoellular carcinomas). (b) Schematic configuration of the automatic data acquisition program (BFS, beat frequency signals and TI, temperature information).

The schematic configuration of multiplexed fiber laser cell temperature-sensing system is shown in Fig. 1(a). The 1480-nm pump laser is transmitted into two fiber laser cavities through a wavelength division multiplexer (WDM) and a 1×2 coupler. The glass fiber laser cavity consists of a pair of FBGs with matched central wavelengths and a piece of EDF. The static FBGs, which are FBG11 and FBG21, are utilized for design of fiber laser cavities and laser modes selection. The FBG12 and FBG22 are two sensing FBGs of two fiber laser cavities, respectively. The two sensing FBGs are contained in glass tubes for cell temperature sensing. MLM lasers will be generated in the fiber laser cavities. Through the 1550-nm port of WDM, those lasers are output to the PD for photoelectric signal conversion to generate multiple BFSs that are modulated by temperature change. RSA and personal computer (PC) are used for signal monitoring and data processing. Note that the two fiber laser cavities have different lengths. From Ref. 19, the BFS’s frequency can be expressed by $v_{N}=Nc/2nL$, where N is the number of BFS, c is the speed of light, n is the effective refractive index of the fiber, and L is the length of fiber laser cavity. Therefore, BFS with different frequencies are generated by those two fiber laser cavities. Temperatures of those two sensing channels can be separately monitored without mutual interference. A platinum resistance thermometer is utilized for cell temperature calibration. It is composed of a platinum resistance (Pt100) and a multimeter (Keysight Type 34461A).

To achieve cell temperature monitoring in real-time, the platinum resistance thermometer and RSA are controlled by Python program to realize automatic data acquisition in seconds. The schematic configuration of the corresponding Python program is shown in Fig. 1(b). To achieve temperature calibration, the corresponding cell temperature is measured by both the fiber laser sensing system and platinum resistance thermometer. The frequency change of a BFS is related with cell temperature change. The frequency change of BFS is recorded by RSA. The corresponding temperature change is recorded by the platinum resistance thermometer. Then the relationship between frequency change of a BFS and temperature change can be determined. The RSA’s parameters including resolution bandwidth, number of sampling points, central frequency, and spectrum bandwidth are determined by Python program. The frequency change of a BFS and temperature change monitored by platinum resistance thermometer can be automatically recorded in real time.

According to the coupled wave theory, the Bragg reflection wavelength $\lambda_{B}$ of the FBG sensor utilized for temperature sensing can be expressed as

$$\lambda_{B}=2n_{\mathrm{eff}}\Lambda ,$$ (1)

where $n_{\mathrm{eff}}$ is the effective refractive index of FBG and Λ is the grating period of the FBG. As the temperature changes, wavelength of the FBG will change

$$\Delta \lambda_{B}=2\Delta n_{\mathrm{eff}}\Lambda +2n_{\mathrm{eff}}\Delta \Lambda =2(\frac{\partial n_{\mathrm{eff}}}{\partial T}\Lambda +n_{\mathrm{eff}}\frac{\partial \Lambda }{\partial T})\Delta T,$$ (2)

where ΔT is the temperature change. Based on Eqs. (1) and (2), the relationship between the FBG sensor’s wavelength and temperature can be expressed as

$$\frac{\Delta \lambda_{B}}{\Delta T}=\lambda_{B}(\frac{\xi }{n_{\mathrm{eff}}}+\alpha ),$$ (3)

where ξ is the temperature coefficient of FBG’s refractive index, and α is the thermal expansion coefficient of fiber.

From Ref. 19, the BFS’s frequency can be expressed by

$$v_{N}=\frac{Nc}{2nL},$$ (4)

where N is the number of BFS, c is the speed of light, n is the effective refractive index of the fiber, and L is the length of fiber laser cavity. Note that MLM lasers are generated in the fiber laser cavity. Its wavelength can be obtained by $\lambda_{m}=2nL/m$,^16^ where m represents the mode number of the laser. Therefore,

$$v_{N}=\frac{Nc}{2nL}=\frac{Nc}{m\lambda_{m}}.$$ (5)

Note that the fiber laser cavity is composed of two FBGs and a piece of EDF. Only the laser modes reflected by the sensing FBG can be excited and transmitted in the fiber laser cavity. Therefore, when $\lambda_{B}$, which is wavelength of the FBG sensor, changes as shown in Eqs. (2) and (3), the MLM laser’s wavelength $\lambda_{m}$ as shown in Eq. (5) will change accordingly.

Therefore, when the temperature of FBG sensor changes, the frequency shift of a BFS can be expressed as

$$\Delta v_{N}=-\frac{Nc}{m\lambda_{m}}\cdot \frac{1}{\lambda_{m}}\frac{\Delta \lambda_{m}}{\Delta T}\cdot \Delta T=-\frac{Nc}{m\lambda_{B}}\cdot \frac{1}{\lambda_{B}}\frac{\Delta \lambda_{B}}{\Delta T}\cdot \Delta T.$$ (6)

Therefore, the wavelength shift of sensing FBG due to temperature change can be demodulated by monitoring frequency change of corresponding BFS as shown in Eq. (6). Using this method, the cell temperature change can be accurately measured by monitoring the frequency change of BFS.

From Eq. (4), it can be seen that the frequency of a BFS is related to the length of fiber laser cavity. By designing two fiber laser cavities with different lengths, BFS with different frequencies are generated by those two fiber laser cavities. Two sensing FBGs that are utilized for cell temperature sensing in two glass tubes can be separately monitored without mutual interference.

**Fig. 2:**
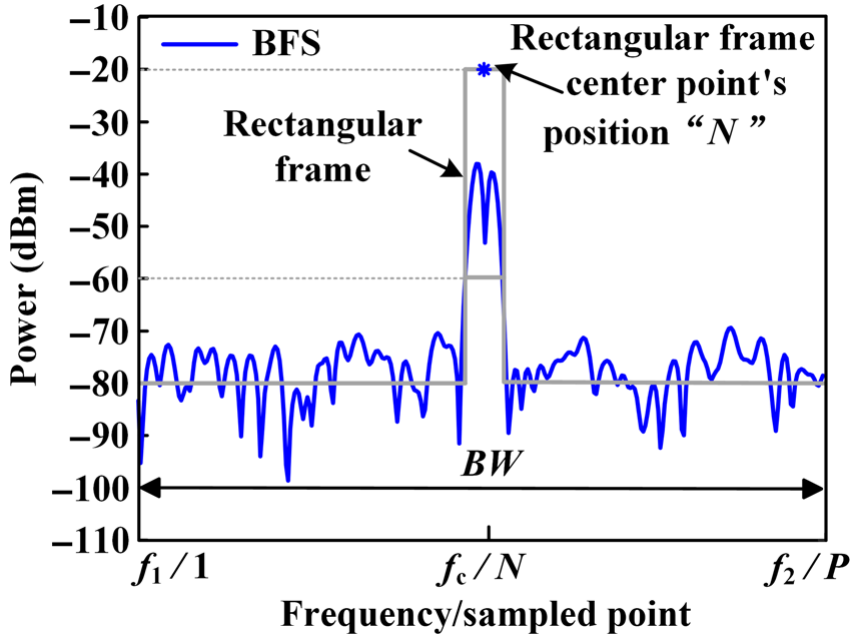
Schematic diagram of the rectangular frame central point demodulation method.

Since multiple BFSs are generated by MLM lasers at PD, there are multiple peaks for BFSs as shown in Fig. 2. When the peak-seeking method is utilized to determine a BFS’s central frequency, large error is easy to be obtained. The sensing system’s accuracy will be affected. Therefore, a central point method is proposed. Schematic diagram of the method is shown in Fig. 2. A rectangular frame is first determined between −60 and −20  dBm. Frequency of the rectangular frame’s central point is determined as frequency of the BFS. Therefore, the BFS’s frequency change can be determined by automatic data acquisition of the rectangular frame’s central frequency.

Since the BFS is obtained by RSA based on digital sampling method, there are P sampled points from frequency $f_{1}$ to $f_{2}$ as shown in Fig. 2. As can be seen, the frequency bandwidth is represented by BW. The first sampled point has the frequency of $f_{1}$. The N’th sampled point has the frequency of $f_{c}$. The frequency of the n’th sampled point can be expressed by

$$(f_{c}-\mathrm{BW}/2)+(\mathrm{BW}/P)*n,$$ (7)

where P is number of the sampled points. According to the one-to-one correspondence relationship between the sampled point and the frequency, the BFS’s frequency change can be accurately monitored. Cell temperature change can be measured based on frequency change of a BFS.

## 3 Materials and Preparation

*Sensing system’s material preparation*. 1480 nm laser (FL-1480 nm-250-B, Max-Ray Photonics, CHN), PD (PIN Detector ET-3500F, Electro-Optics Technology, United States), 1480/1550  nm WDM (WDM-1X2-980/1550-1-A72, Max-Ray Photonics, CHN), 1×2 coupler, RSA (RSA306B, Tektronix, United States), PC with Python automatic data acquisition program, two fiber laser cavities with different lengths, multimeter 34461A keysight, platinum resistance Pt100, and several connecting cables.

*Experimental preparation*. Dulbecco’s minimal essential medium (DMEM) and fetal bovine serum are from Gibco (Presley, Scotland, and United Kingdom), phosphate buffer solution is purchased from Beyotime Biotechnology Co., Ltd. (Nantong, China).

*Preparation of complete cell culture solution*. 45 mL DMEM culture solution is added into 50 mL centrifuge tube. 5 mL bovine serum is also added. Those solutions are mixed evenly and set aside.

*HepG2 cell solution*. 1 mL of trypsin is added to the culture dish with cells. It is gently shaken to cover the bottom of the dish. Then the culture dish is moved into the incubator. After digested for 2 min and then taken it out. 1 mL of complete culture solution is added to stop the reaction of trypsin and all the cells are blown down with a liquid transfer gun. The cells are transferred to a 5-mL centrifuge tube and then centrifuged at 800 rpm for 5 min. The supernatant liquor is discarded and 2 mL of complete culture solution is added. Subsequently, 10  μL of the above cell-containing culture solution was added to the cell counting plate and counted under a microscope. Finally, the cell stock solution was diluted using DMEM cell culture solution containing 10% bovine serum protein at a certain dilution multiple to bring the concentration of the diluted cell solution to $1\times 10^{6}\text{ cells}/\mathrm{mL}$, thus ensuring a consistent number of cells for each experiment.

AFB1 is purchased from Sigma-Aldrich Company. AFB1 powder is first dissolved in dimethyl sulfoxide to prepare 0.2  mg/mL stock solution and stored at −20°C away from light, then diluted with cell complete culture solution to the concentration used for experimental analysis.

## 4 Experiment and Discussions

**Fig. 3:**
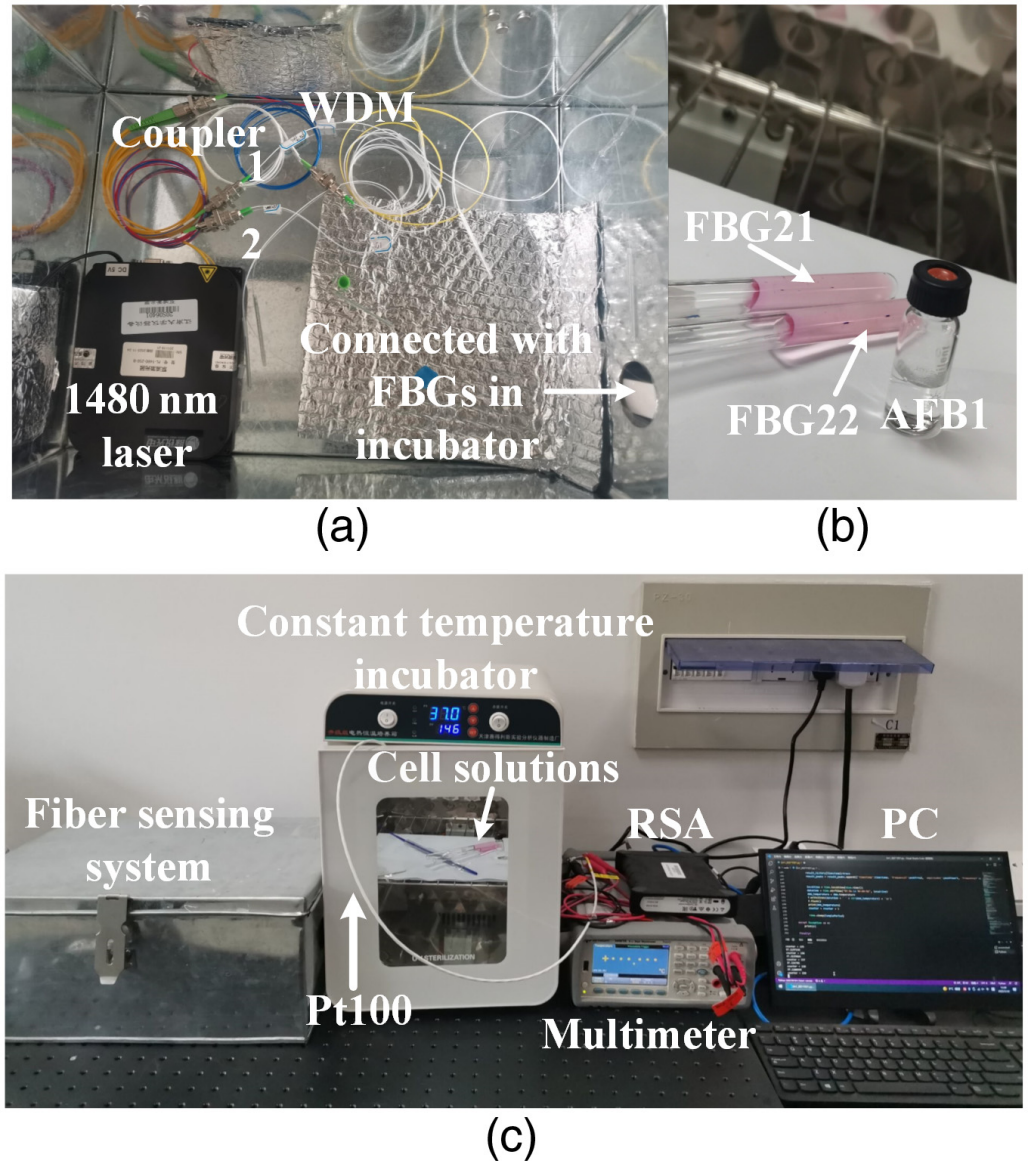
Test environment and instruments: (a) fiber sensing system; (b) FBG sensors utilized for HepG2 cell temperature sensing; and (c) the test environment.

Experimental setup of the optical fiber sensor system is shown in Fig. 3. The 1480-nm pump laser is transmitted into two fiber laser cavities through a WDM and a 1×2 coupler. The glass fiber laser cavity consists of a pair of FBGs with matched central wavelengths and a piece of EDF. The FBG sensors are common commercially acquired. MLM lasers will be generated in the fiber laser cavity. The static FBGs that are FBG11 and FBG21 are utilized for design of fiber laser cavities and laser modes selection. The FBG12 and FBG22 are two sensing FBGs of two fiber laser cavities, respectively. The two sensing FBGs are in glass tubes for cell temperature sensing. The lengths of sensing FBG sensors are about 1 cm. The diameter of fiber is about 200  μm. Therefore, the sensing FBG sensor has a very compact size. The static FBGs are attached in the experimental metal box. For the fiber laser cavity, the whole cavity is surrounded by heat insulating material. Only the sensing FBGs are immersed in the HepG2 solutions. Therefore, the temperature changes of HepG2 solutions can be accurately measured. The lengths of two glass fiber laser cavities are 1.3 and 1.34 m, respectively. The EDF has an absorption coefficient of 37.8  dB/m at 1532 nm. The lengths of EDFs are 0.65 and 0.7 m, respectively. The corresponding parameters of four FBG sensors are shown in Table 1. The FBG sensor’s 3-dB bandwidth is measured by OSA YOKOGAWA AQ6370D, which has a wavelength accuracy of ±10  pm. The sensing FBG12 and FBG22 are in two glass tubes, respectively. Those are utilized to measure the temperature changes of HepG2 cell solutions as shown in Fig. 3(b). The 1480-nm pump laser, WDM, and 1×2 coupler are placed in an experimental metal box as shown in Fig. 3(a). The temperature fluctuation caused by surrounding environment can be avoided. The two glass tubes with HepG2 cell solutions are placed in an incubator with constant temperature. The platinum resistance Pt100 as shown in Fig. 3(c) is placed in the incubator. Through the 1550-nm port of WDM, MLM lasers are output to the PD for photoelectric signal conversion to generate multiple BFSs. The BFS are monitored by the RSA. Corresponding frequency changes of BFSs are automatically recorded by Python program. As the same time, the multimeter (Keysight Type 34461A) is connected with PC and the temperature change is automatically recorded by Python program. During the whole experiment, the incubator keeps a constant temperature.

**Table 1:** Parameters of four FBGs.

	3-dB bandwidth (nm)	Central wavelength (nm)	Reflectivity (%)
FBG11	0.22	1548.00	92.92
FBG12	0.22	1547.94	92.06
FBG21	0.23	1549.91	91.09
FBG22	0.22	1549.96	92.06

**Fig. 4:**
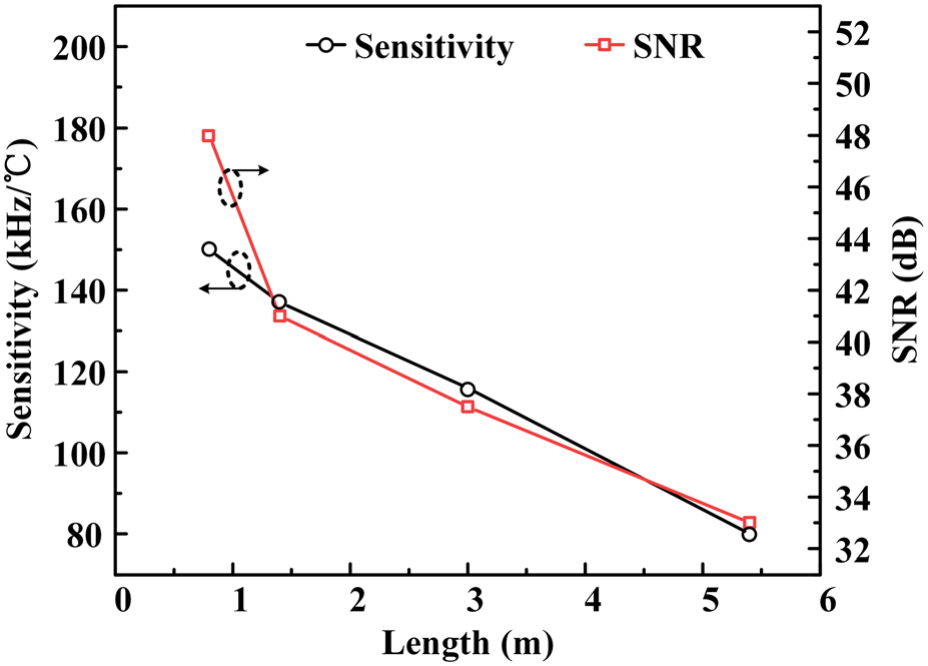
BFS’s temperature sensitivity and SNR increase as the fiber laser cavity’s length reduces.

In order to achieve a high-sensing sensitivity and accuracy, the BFS’s temperature sensitivity and signal-to-noise ratio (SNR) are researched. As can be seen from Fig. 4, the length of the whole fiber laser cavity is changed by reducing the length of FBG sensor’s fiber pigtail. The length of EDF is unchanged. At first, the length of the whole fiber laser cavity is about 5.4 m. The temperature change is applied on the sensing FBG sensor. Then corresponding temperature sensitivity is measured. The SNR of corresponding BFS at about 380 MHz is also measured. Then the fiber pigtails of FBG sensors are reduced. Length of the fiber laser cavity is changed from 5.4 to 0.8 m as shown below. Both the temperature sensitivities and SNRs at different cavity lengths are measured. As can be seen, the BFS’s temperature sensitivity and SNR increase as the fiber laser cavity’s length reduces. Therefore, the lengths of two fiber laser cavities utilized in this experiment are designed to be about 1.3 m. Performance requirement and easy to operate are both considered.

**Fig. 5:**
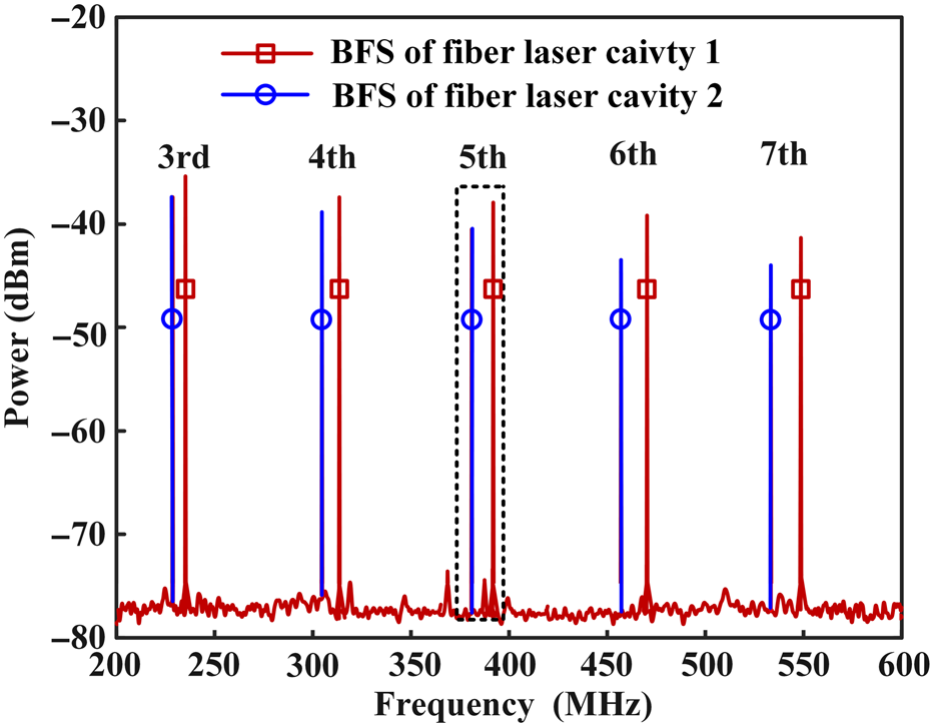
BFSs generated by two fiber laser cavities.

BFSs are monitored by RSA. As can be seen from Fig. 5, BFSs generated by different fiber laser cavities are shown in different colors. Five BFSs of each fiber laser cavity are displayed by the RSA. As can be seen, the frequencies of the fifth BFSs are about 381 and 391 MHz, respectively. Those two BFSs are utilized for temperature sensing of two channels.

**Fig. 6:**
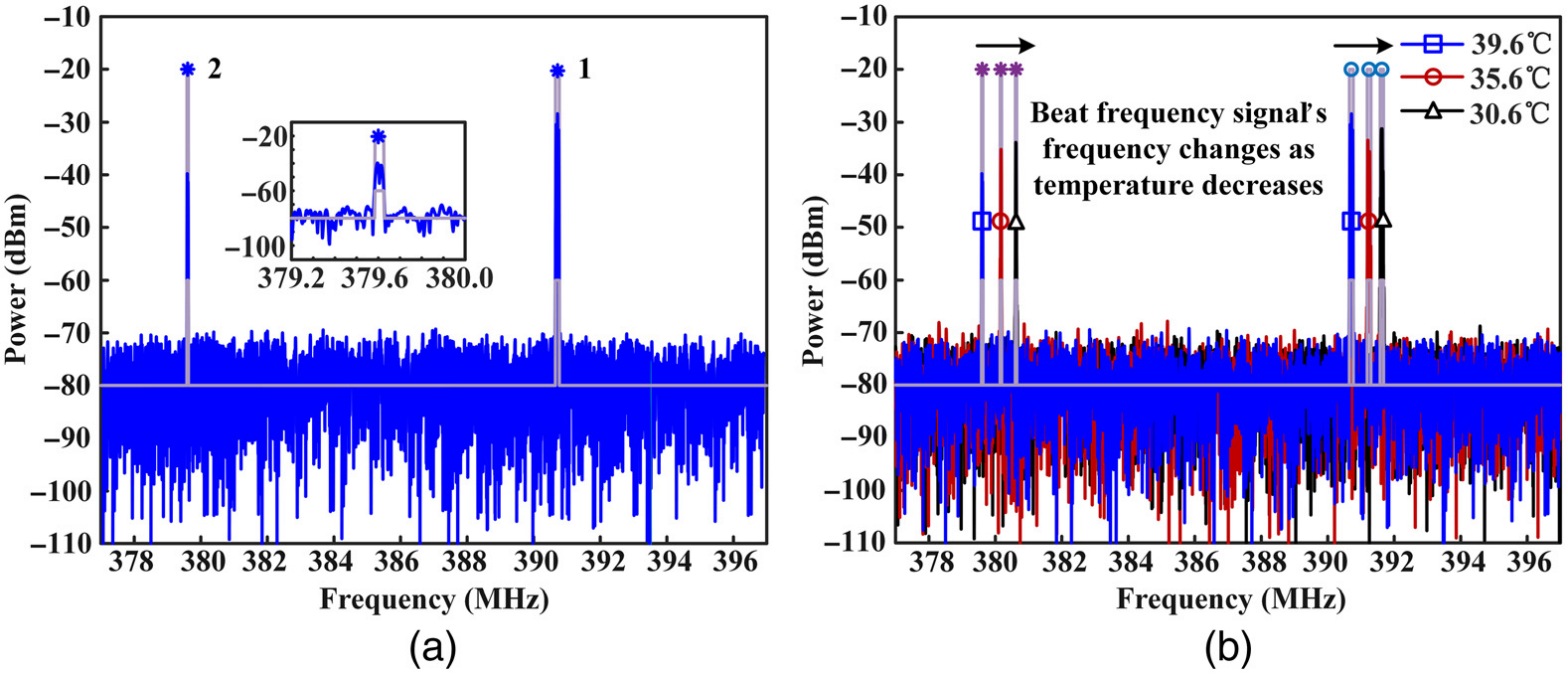
(a) Two BFSs of two fiber laser cavities at 39.6°C. (b) Frequencies of BFSs increase as the water’s temperature decreases from 39.6°C to 30.6°C.

Temperature calibration is first operated to improve measurement accuracy of the sensing system. The FBG12 and FBG22 are placed in a glass tube with water. At the same time, the water’s temperature is also monitored by platinum resistance thermometer. As can be seen from Fig. 6, the water’s temperature is 39.6°C. The frequencies of BFSs are at 381 and 391 MHz corresponding to fiber laser cavity 1 and fiber laser cavity 2. The central points of BFSs are marked with asterisks as shown in Fig. 6(a). As the water’s temperature decreases, frequencies of BFSs increase as shown in Fig. 6(b).

**Fig. 7:**
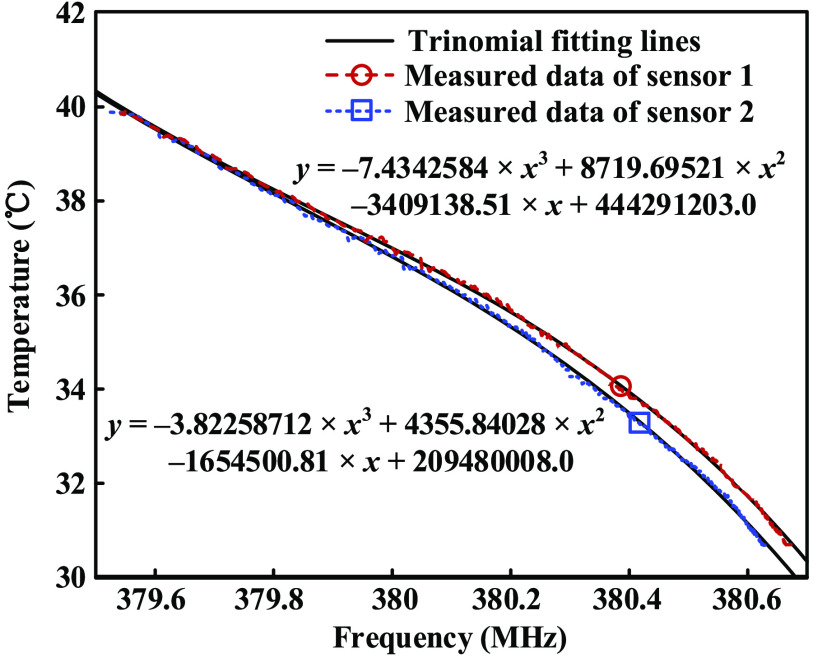
Trinomial fitting of the relationship between BFS’s frequency and temperature.

Since the water’s temperature is also monitored by platinum resistance thermometer, the relationship between BFS’s frequency and temperature can be determined as shown in Fig. 7. The tested average temperature sensitivities of sensor 1 and sensor 2 are 101.62 and 119.82  kHz/°C, respectively.

**Fig. 8:**
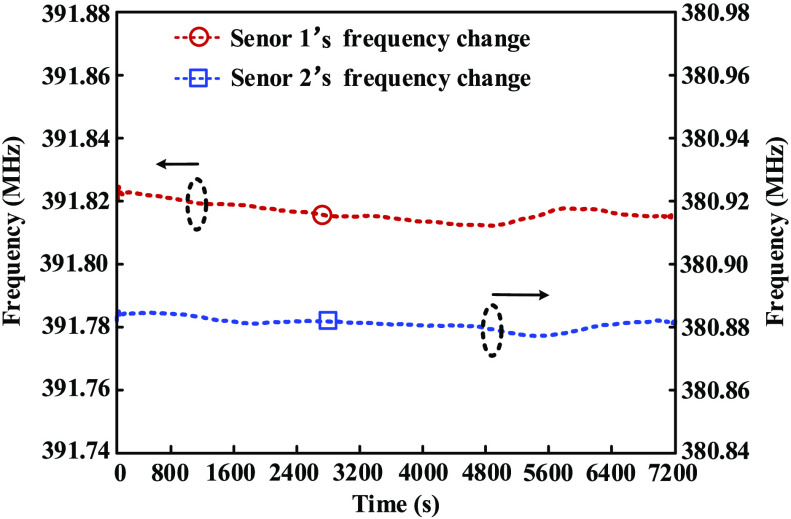
Temperature stability test of the sensing system at room temperature.

In addition, the stability of the fiber sensing system is tested. The measurement is carried out for totally 120 min. As can be seen from Fig. 8, the maximum frequency fluctuations of sensor 1 and sensor 2 are about 12.5 and 7.8 Hz, respectively. Since the temperature sensitivities of both sensors are higher than 100.0  kHz/°C, the tested temperature fluctuations of both sensors at room temperature are smaller than 0.01°C which demonstrated the high stability of the designed sensing system.

After the fiber sensing system’s performances are demonstrated, the temperature change of human HepG2 cells under the influence of exogenous chemical AFB1 is monitored by the sensing system. The HepG2 cells are cultured at a constant temperature of 37°C. First heat the incubator to 37°C and keep its temperature unchanged. 0.9 mL AFB1 with a concentration of 0.2  mg/mL is put into one glass tube. The toxins are placed in the constant temperature incubator during the experiment. Then 1.8 mL HepG2 cell solution is taken from cell chamber and is transferred to glass tube 1 and glass tube 2 by a liquid transfer gun. 0.9 mL is for each tube. The FBG12 of sensor 1 is put into glass tube 1 and the FBG22 of sensor 2 is put into glass tube 2. Make sure the sensing FBGs are completely immersed in the cell solutions. Then the sensing system begins to automatically monitor the HepG2 cell solutions’ temperatures for 5000 s. In order to make sure the temperatures of HepG2 cell solutions in two glass tubes are the same before AFB1 solutions are added to the cell solutions, the two glass tubes are first placed in the incubator for 40 min. After 40 min, the cell solutions and toxin’s temperatures are the same. Then 0.2 mL AFB1 solution is transferred from the toxin tube to glass tube 2 by a syringe. 0.2 mL AFB1 solution is also injected into glass tube 1 with cell solution. Note that the transfer of AFB1 solution is operated in the incubator and the sensing FBGs are always immersed in the cell solutions. The temperatures of both glass tubes with cell solutions are automatically monitored for about 1.5 h.

**Fig. 9:**
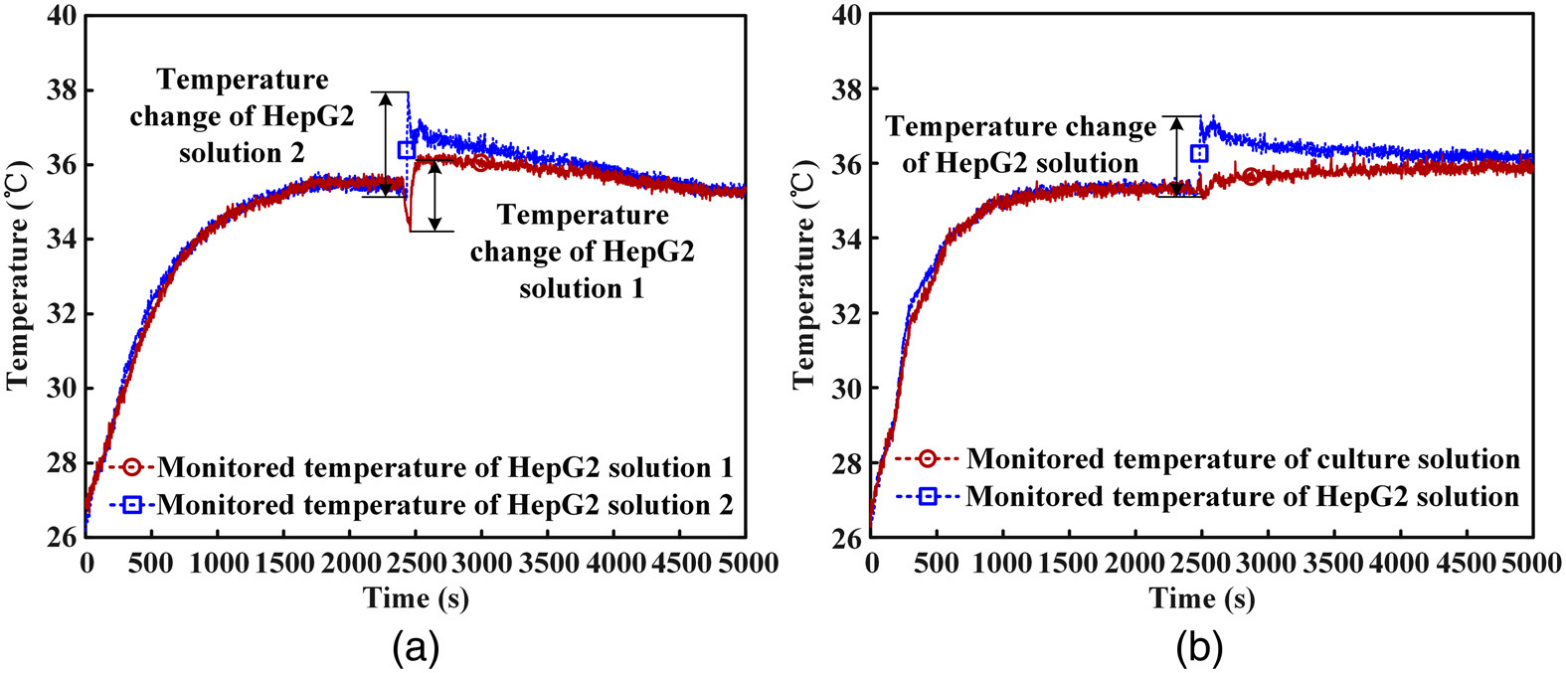
(a) Temperatures of HepG2 cell solutions increase under the influence of AFB1. (b) Temperature of HepG2 cell solution increases about 2.0°C while the temperature of culture solution is almost not changed.

The tested temperature changes of HepG2 cell solutions under the influence of AFB1 are shown in Fig. 9(a). It can be seen that the temperatures of HepG2 cell solutions at the beginning of temperature monitoring are about 26.5°C. After the temperatures of cell solutions are monitored for about 40 min, the AFB1 solutions are added to HepG2 cell solutions. The temperature of cell solution in the glass tube 1 changes 2.01°C. The temperature of cell solution in the glass tube 2 changes 2.83°C. As can be seen, the cell solutions’ temperatures decrease before AFB1 solutions are added. This is because the incubator’s door opens for toxins transfer. After the toxins are transferred, the incubator’s door closes again. From Fig. 9(a), it can be seen that the temperatures of HepG2 cell solutions increase when AFB1 solutions are added.

To further demonstrate the temperature increase effect of HepG2 cells under the influence of exogenous chemical AFB1, temperature change of culture solution under the influence of AFB1 solution is monitored. As a comparison, temperature change of cell solution under the influence of AFB1 is also monitored. The experiment process is similar to the aforementioned experiment. First heat the incubator to 37°C and keep the temperature unchanged. Then 0.9 mL culture solution and 0.9 mL HepG2 cell solution are taken from the cell chamber. The culture solution is transferred to a glass tube 1 by a liquid transfer gun and the HepG2 cell solution is transferred to a glass tube 2. Put the FBG12 of sensor 1 into the cell solution and put the FBG22 of sensor 2 into the culture solution. Make sure, the sensing FBGs are completely immersed in the solutions. 0.9 mL AFB1 solution with a concentration of 0.2  mg/mL is put into one glass tube 3. The toxins are placed in the constant temperature incubator during the experiment. After 40 min, 0.2 mL AFB1 solution is transferred from the toxin tube to cell solution by a syringe. 0.2 mL AFB1 solution is also injected into culture solution. The temperatures of both solutions are automatically monitored for about 1.5 h. The experimental environment and settings are exactly the same with the aforementioned experiment.

The temperature changes of both solutions during the whole experiment are monitored by the sensing system as shown in Fig. 9(b). When AFB1 solutions are added, the temperature of HepG2 cell solution increases about 2.0°C while the temperature of culture solution is almost not changed. This also demonstrates the temperature increase effect of HepG2 cells under the influence of exogenous chemical AFB1.

## 5 Conclusion

In this paper, a multiplexed optical fiber cell temperature sensing system has been presented with detailed design procedure and analysis. To the best of the authors’ knowledge, this is the first time an MLM optical fiber laser sensor array is utilized for cell temperature sensing. Two glass fiber laser cavities with lengths of 1.31 and 1.34 m are utilized for two-channel sensing and both channels can be monitored in real time. The EDF has an absorption coefficient of 37.8  dB/m at 1532 nm. The lengths of EDFs are 0.65 and 0.7 m, respectively. A pair of common commercial FBGs with matched central wavelengths about 1550 nm can be utilized for MLM lasers generation. From both theory and experiment, the BFS’s temperature sensitivity and SNR increase as the fiber laser cavity’s length reduces. By utilizing Python program to control platinum resistance thermometer and RSA, the BFS’s frequency shift that represents temperature change can be automatically recorded in seconds. In order to achieve an accurate demodulation of BFS’s frequency shift, a method of finding the BFS’s center point is utilized, which can avoid the error caused by large frequency jitter. By utilizing the designed sensing system, the temperature change of HepG2 cells under the influence of exogenous chemical AFB1 can be monitored in real time. This study provides a simple and effective method to design a fiber laser sensor system without complex demodulation techniques and expensive optical components. The sensing system can easily be designed to have multiple sensing channels by simply increasing the number of fiber laser cavities. It has great value for the temperature monitoring of different cell solutions under the influences of different exogenous chemicals in real time.
